# Accelerated aspiration with Q™ catheter: An *in vitro* study

**DOI:** 10.1177/15910199241273974

**Published:** 2024-11-06

**Authors:** Philippe Reymond, Mayra Contreras, Olivier Brina, Trent Langston, Naomi Chesler, Waleed Brinjikji, John Wainwright, Paolo Machi

**Affiliations:** 1 Division of Diagnostic and Interventional Neuroradiology, Geneva University Hospitals, Geneva, Switzerland; 2 Mivi Neuroscience, Eden Prairie, MN, USA; 3 Samueli School of Engineering at University of California Irvine, Irvine, CA, USA; 4 Radiology, Mayo Clinic, Rochester, MN, USA; 5 Neurosurgery, Mayo Clinic, Rochester, MN, USA

**Keywords:** Acute ischemic stroke, catheters, *in-vitro* methods

## Abstract

**Background and Purpose:**

Thrombectomy in distal, medium vessels is a topic of increasing interest. To date, there are few *in vitro* studies focused on performance of ≤5F catheters in medium vessels. The purpose of this study is to compare the performance of the 3F, 4F, and 5F MIVI Neuroscience Q Catheters versus Penumbra 3F, 4F, and MicroVention Sofia 5F Catheters.

**Methods:**

Using *in vitro* methods, we assessed and compared the following parameters: aspiration flow rates, clot uncorking forces, impulse, and clot ingestion. For flow rate, each aspiration catheter was immersed in a cylindrical container. Flow rate at one second was used to calculate impulse. For clot uncorking force, the force required to disengage a catheter from a simulated clot was recorded. For ingestion, we measured time to ingest soft and medium stiffness synthetic clots.

**Results:**

The measured flow rates without a stent retriever for the Q3, Q4, and Q5 catheters were 3.54 ml/s, 5.32 ml/s, and 6.87 ml/s. The measured flow rates without a stent retriever for the 3MAX, 4MAX, and 5F Sofia were 1.46 ml/s, 2.56 ml/s, and 1.73 ml/s. The impulse calculated for one second was 26 mNs for Q5 vs 9 mNs for Sofia 5, 35 mNs for Q4 vs 15 mNs for 4Max< and 35 mNs for Q3 vs 9 mNs for 3Max. The average system ingestion for Q was significantly faster than the competitive catheters.

**Conclusions:**

The Q catheters demonstrated higher flow rates, higher uncorking force, and faster complete clot ingestion than competitive catheters.

**Table:**
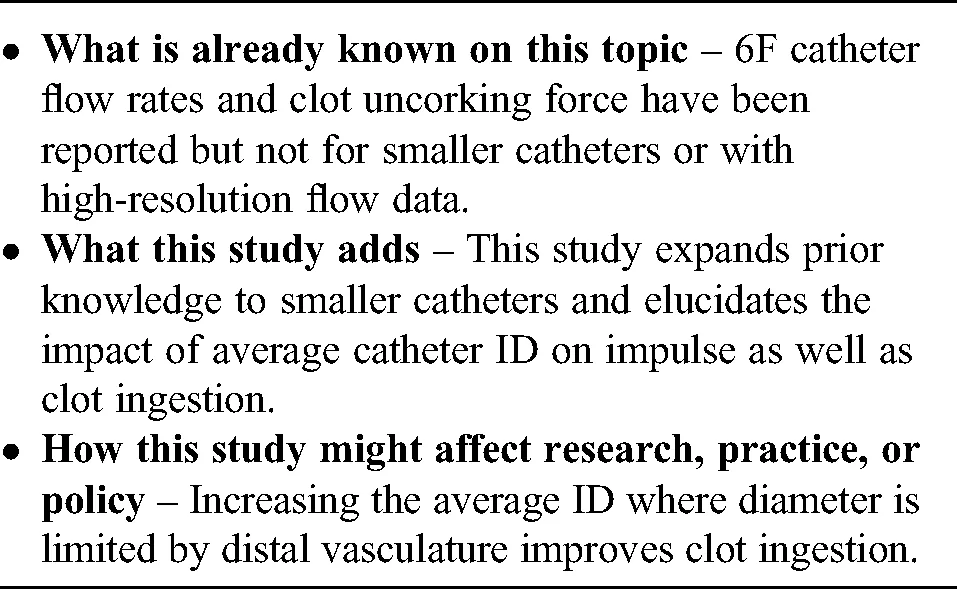

## Introduction

Mechanical thrombectomy using contact aspiration is widely performed in patients with large vessel occlusions (LVOs) and distal and medium vessel occlusions (DMVOs) among patients with acute ischemic stroke. There have been extensive benchtop studied evaluating the efficacy of various catheter systems for contact aspiration of LVO,^1–3^ considerably less has been published evaluating the *in vitro* efficacy of aspiration systems for DMVO.

*In vitro* studies evaluating the efficacy of various aspiration systems in the treatment of DMVO are important for several reasons. First, understanding the physical principles affecting clot engagement and clot ingestion is important as it allows us to develop a better understanding of how contact aspiration works and how it can be improved. Second, *in vitro* studies allow us to understand failure mechanisms of contact aspiration systems which can also help better inform technique and catheter design.

The Q Aspiration System from MIVI differs substantially from other aspiration catheters in that the Q catheter is an extension catheter which is delivered through a 0.088″–0.090″ guiding sheath. While the Q3, Q4, and Q5 catheters have similar tip ID and OD to competitive 3–5F catheters, respectively, the Q catheters benefit from the larger proximal ID of the guiding sheath resulting in a large effective ID (see Figure 1(a)). This is particularly important for DMVO in that aspiration catheters are generally well-matched to vessel size so increases in catheter tip ID for full length 3–5F aspiration catheters will be incremental at best.

**Figure 1.**
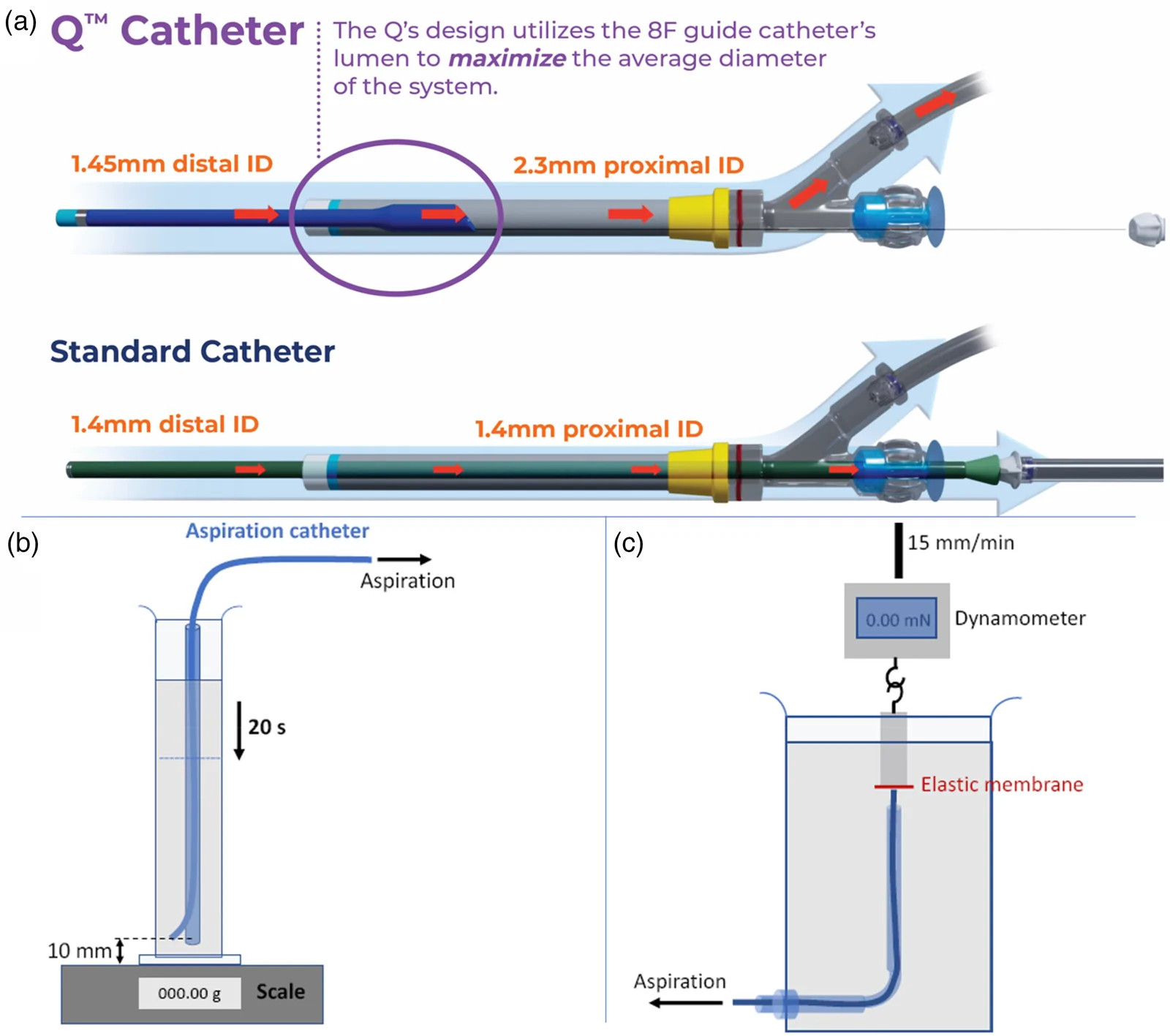
Schematic illustrations of (a) Q5 aspiration catheter with its single point of aspiration, sliding seal, and extension catheter design vs standard 5F aspiration catheter. (b) The set-up for flow rate measurement. Pressure on the distal tip of the catheter is higher than atmospheric pressure due to the high-water column but much lower than arterial pressure. (c) The set-up for clot uncorking force which is the force to dislodge a corked thrombus from aspiration catheter's distal tip as the catheter is retrieved.

The purpose of this study is to perform an *in vitro* study comparing the MIVI Q3, Q4, and Q5 catheters to commercially available full length aspiration catheters with 3F, 4F, and 5F IDs. In particular, we compared flow rates, impulse, clot uncorking force, and clot ingestion between the Q system and full length 3–5F catheters. Additionally, the impact of stent retriever usage on flow rates in these smaller distal tip catheters is explored.

## Methods

### Flow rate test with and without stent retriever

Flow rate was measured as previously described.^4^ Briefly, each aspiration catheter distal segment was immersed at 1 cm from the bottom of a cylindrical container (initial water height 11.5 cm) filled with 1.4 liters of water at 20°C and placed on a precision balance (Figure 1(b)). The aspiration catheter was connected to a vacuum system (Penumbra Inc, Alameda, CA, USA). When the pump reached a maximum negative pressure of −87 kPa, the circuit was unclamped. The volume of water aspirated in 20 s was measured to obtain the flow rate (ml/s). The experiments were repeated using a Solitaire 4 × 20 mm (Medtronic, Galway, IR) was placed just distal to the tip of the aspiration catheter with a wire diameter of 0.018″.^5^ The microcatheter was removed during this test. Three test runs were performed with each device.

### Peak flow rate and impulse

A flow sensor (ILT Reinach, Switzerland) with a 50 Hz data collection frequency was used with a vacuum pump set to −29inHg, 37°C for 30 s using water as media. Peak flow rate and flow at one second were determined using MATLAB (Mathworks Natick, MA). Peak flow rate was averaged from three trials per catheter. The peak flow at 1 s was used to measure the impulse value per equation below for all of the catheters. Impulse (J) is the change in momentum ( Δp ) per unit time. We measure the initial impulse applied by the catheter at the tip by computing the mass of the water in the catheter (m; in g) multiplied by the difference in fluid velocity from the beginning of aspiration (t = 0 s; v_t = 0 _= 0 m/s) to 1 s (v_t = 1s_). One second was chosen because it is before constant flow is reached. Therefore, this initial impulse $J_{1\sec}=\Delta p_{1\sec}=m(v_{t=1s}-v_{t=0s})=m(v_{t=1s})$

### Clot uncorking force

The test was conceived to evaluate the force to dislodge a corked thrombus from aspiration catheter's distal tip as the catheter is retrieved. By using a polymer membrane that mimics clot stiffness, a more clinically relevant measurement can be obtained than a simple cross-sectional area times pressure. Uncorking force was measured three times for each catheter as described previously,^4^ inside a container filled with water (Figure 1(c)). Once the vacuum system was activated and a maximum negative pressure of −87 kPa was reached, the cylinder to which the elastic membrane has been applied was withdrawn by the traction machine at a constant speed of 15 mm/min. The force was then recorded by data acquisition software over time. The value corresponding to the moment of detachment between the elastic membrane and the aspiration catheter was considered as the clot uncorking force.

### Clot ingestion test

Clear silicone tubes were used to simulate brain vessels. Synthetic soft and medium clots (Life Model Designs, Marengo, OH) were used to represent red blood cells dominant and fibrin-rich clot, respectively. Clot diameter was ∼0.5 mm larger than designated tube ID to create adherence and the tube ID was ∼0.5 mm larger than the French size being tested. Each set of tests were repeated four times per clot type per catheter. The clot length was cut to challenge each catheter size (range 14–32 mm) and kept within 1 mm for each set of eight comparison tests. Clot ingestion was taken as the time for the clot to reach the canister. The test was stopped at 300 s and the amount of clot distal to the tip (corked) was measured. Additionally, a representative video using florescent clot was obtained with the same setup so the clot ingestion rate could be visualized (see supplemental video).

### Statistical analysis

Excel Version 2312 was used to calculate Students’ t-test and p-values reported.

## Results

### Flow rate

Flow rate tests with and without a stent retriever are presented in Figure 2. The measured flow rates without a stent retriever for the Q3, Q4 and Q5 catheters were 3.54 ± 0.01 ml/s, 5.32 ± 0.06 ml/s, and 6.87 ± 0.03 ml/s, respectively. The measured flow rates with a stent retriever were 2.2 ± 0.06 ml/s, 3.65 ± 0.01 ml/s, and 5.39 ± 0.04 ml/s, respectively. The measured flow rates without a stent retriever for the 3MAX, 4MAX, and 5F Sofia were 1.46 ± 0.02 ml/s, 2.56 ± 0.02 ml/s, and 2.91 ± 0.03 ml/s, respectively. The measured flow rates with a stent retriever were 0.76 ± 0.02 ml/s, 1.73 ± 0.03 ml/s, and 2.05 ± 0.03 ml/s, respectively (Figure 2(a)). Figure 2(b) illustrates that the Q Catheter has a higher peak flow rate and reaches the peak flow rate faster than similar sized competitive catheters. In all cases, the Q Catheter flow rate was statistically higher than the competitive catheter p < 0.001.

**Figure 2.**
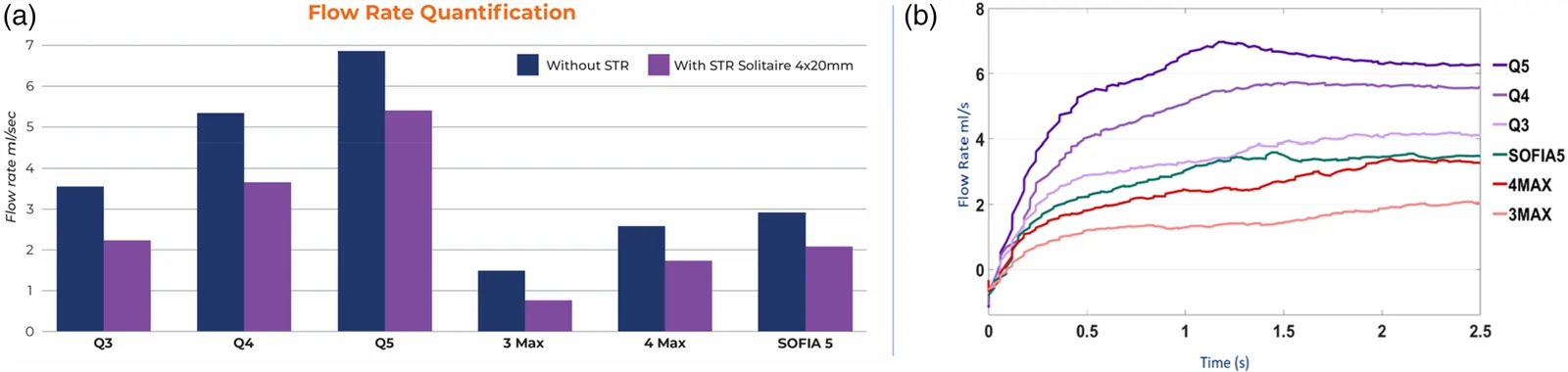
(a) Flow rate tests with and without a stent retriever. (b) Flow rate vs time graph.

### Impulse and uncorking force

The impulse calculated for one second was 26 ± 1.3 mNs for Q5 vs 9 ± 1.9 mNs for Sofia 5, 35 ± 1.2 mNs for Q4 vs 14 ± 0.6 mNs for 4Max, and 36 ± 1.8 mNs for Q3 vs 8 ± 1.4 mNs for 3Max. The simulated uncorking force of the Q3 was 44.0 ± 1.0 mN and 3 Max was 32.3 ± 2.3 mN, Q4 was 60.3 ± 2.9 mN and 4 Max was 46.7 ± 3.1, and Q5 was 104.3 ± 9.3 mN and SOFIA 5 71 ± 6.4 mN. In all cases, the Q catheter impulse and uncorking force were statistically higher than the competitive catheter p < 0.005 (see Figure 3).

**Figure 3.**
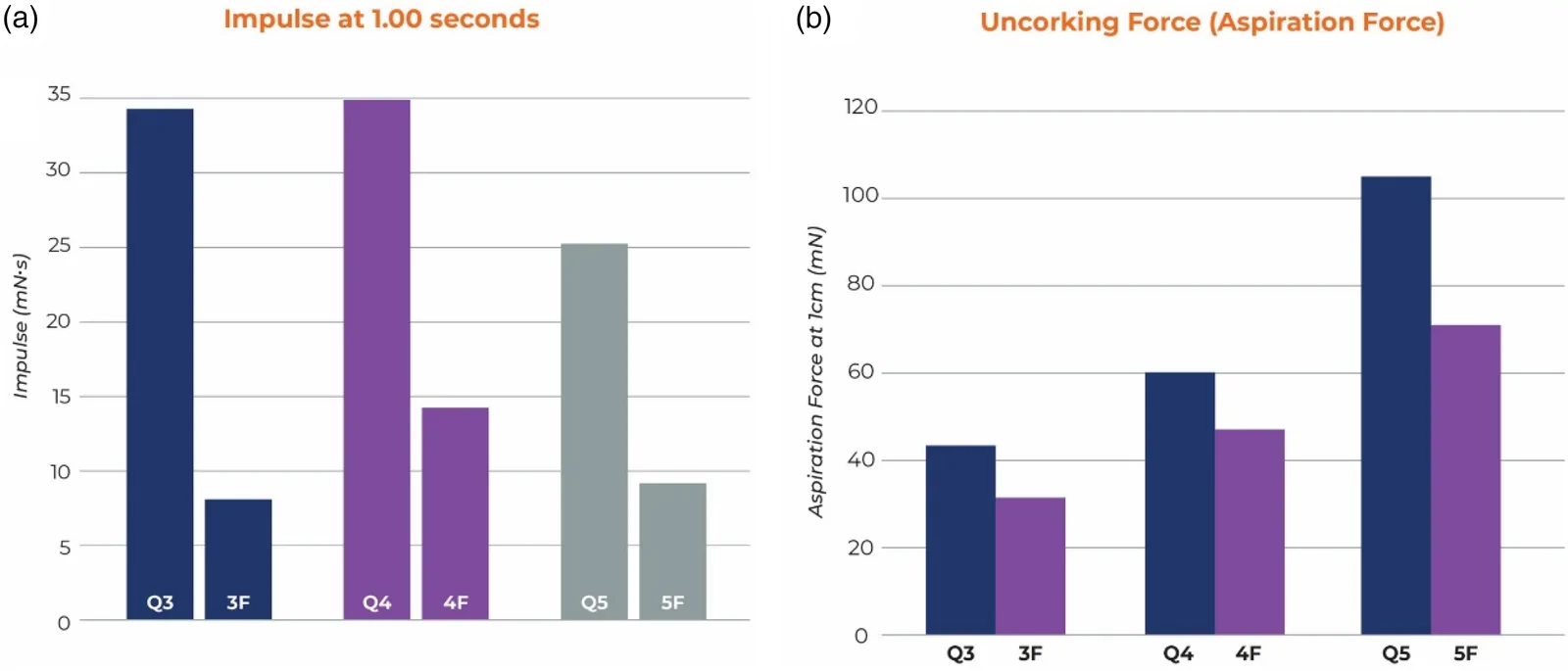
(a) Impulse at 1 s and (b) uncorking force (aspiration force) comparison.

### Ingestion

The average system ingestion for Q3 was 30 ± 2 s for soft clot and 44 ± 5 s for medium clot whereas for 3Max it was >300 s for all soft clots and 271 ± 54 s for medium clot. For Q4, ingestion took 10 ± 1 s for soft clot and 21 ± 6 s for medium clot but 4Max took 274 ± 53 s for soft and >300 s for all medium clots. For Q5, ingestion took 77 ± 11 s for soft clot and 31 ± 2 s for medium, however, Sofia 5 took more than 300 s for all soft and firm clots. Sofia 5 had an average of 19 mm of medium clot distal to the tip at the end of 300 s. The 4Max had 5 mm of medium clot distal to the tip. The 3 Max had 2 mm of soft clot and 8 mm of medium clot corked distal to the catheter tip. In all cases, the Q system clot ingestion was complete and statistically faster than the competitive catheter p < 0.001 (see Figure 4).

**Figure 4.**
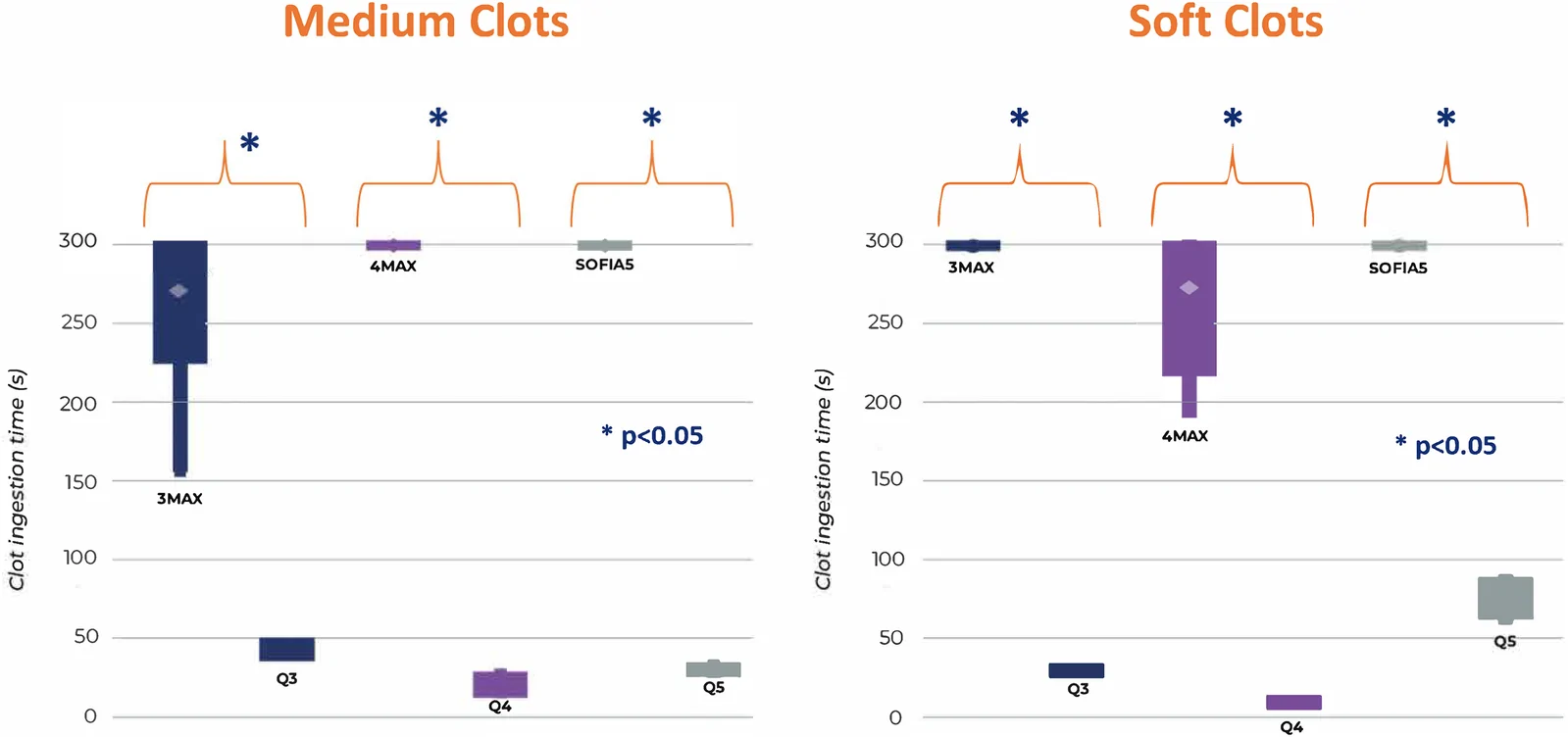
Clot ingestion time for Q system was significantly faster than the competitive catheters *p value <0.001. Time was stopped at 300 s and the remaining clot outside the catheter was measured.

## Discussion

Our study comparing flow rate, impulse, uncorking, and clot ingestion rates of the Q3-5 system versus competitive full-length catheters demonstrated a number of interesting findings. First, we found that the extension catheter design of the Q system was associated with significantly higher flow rates compared to full length 3–5F catheters both without and with a stent retriever. Second, we found that impulse due to peak flow was also higher with the Q3-5 system compared to full length 3–5F catheters. Third, clot uncorking force was roughly 50–60% higher for Q3–5F systems compared to full length 3–5F catheters. Lastly, and most importantly, clot ingestion was faster and more complete with the Q system than the full-length catheters. These findings are important as they support the hypothesis that extension catheters which leverage proximal IDs from guiding sheaths resulting in higher flow rates could potentially allow for superior recanalization due to a combination of physical principles resulting in superior clot ingestion.

Metrics used to assess catheter performance typically include flow, force, and clot ingestion.^3^ Since the pressure gradient driving flow is uniform across catheter designs, the maximum flow depends on the total shear stress in the catheter, often computed assuming fully developed laminar flow using the Hagen–Poiseuille law such that shear stress is proportional to the inverse of radius to the third power. Force generated at the tip of the catheter is the product of the vacuum pressure and the cross-sectional area of the tip. Therefore, maximizing the average radius of the catheter and catheter tip for the vessel size being treated maximizes both flow and force. However, when it comes to distal vessels (M2 and beyond), currently available aspiration systems are already almost completely matched to the target vessel diameter. This presents a problem for aspiration catheter development as newer full length aspiration catheters can only achieve incremental ID improvements given the already maximized OD for these target vessels. A novel approach is to increase proximal catheter size while keeping distal catheter ID and tip cross-sectional area small. This approach reduces shear stress, which increases flow, and maintains catheter force as demonstrated in our experiments. Here, we also introduce a new metric to assess aspiration catheter performance: impulse. Impulse is the change in momentum computed as the integral of force over time. As shown by equation (1), a greater mass of fluid in the catheter increases impulse, as does greater acceleration of flow at the tip. An analogy is hitting a golf ball with a golf club; for a given size club and ball, swinging the club faster generates higher impulse. Similarly, for a hammer and nail, a faster swing drives the nail into the wood further. Due to conservation of momentum, the impulse generated by the catheter acts on the clot at the catheter tip, which works to dislodge the clot from the vessel wall and ingest the clot. For the same tip diameter, higher flow rates (generated with a larger average ID of a catheter as in the Q catheters) increase both the mass of fluid in the catheter and generate faster acceleration, which significantly increases the change in momentum and results in better clot ingestion.

Because combination therapy (aspiration plus stent retriever) is so commonly used, we also studied flow rate with a stent retriever inside the catheter. Because the effective average ID subtracts out the cross-sectional area that the stent retriever wire or microcatheter occupies, aspiration flow is significantly impacted by the presence of a stent-retriever, especially for smaller catheter systems. As a percent reduction, starting with a larger average ID reduces the flow relatively less and is the reason the Q catheter flow is impacted by the stent retriever less. Conversely, the microcatheter is often removed before retraction of the thrombectomy system as this increases the effective average ID.^6^ The percentage ID reduction of the stent wire on flow is less as the ID grows but impacts catheters of all sizes. Therefore, the extension catheter design of the Q system will provide an accelerated flow benefit regardless of size.

These *in vitro* benefits of the Q catheter also appear to translate into clinical data. Recently, a Q MeVO multi-center international experience showed a mFPE of 70%,^7^ whereas a MeVO systematic review only had a mFPE of 51%.^8^ Similarly, final Q catheter mTICI ≥ 2b was 85% but was only 77% in the systematic review. This trend needs to be verified with further clinical studies.

Limitations of this study are that it only included *in vitro* testing. The clots used were synthetic however they are consistent within and between batches and commercially available so testing can be repeated easily. Also, we did not study outcomes in a tortuous model but concentrated on elucidating the mechanism and effect of a larger effective ID. Saline not blood was used in the flow experiments, but the same trends would be seen regardless of viscosity.

## Conclusion

These results highlight the significant advantages of the Q3-5 catheters versus 3MAX, 4MAX, SOFIA5 in terms of flow rate, impulse, clot uncorking force, and clot ingestion. *In vitro*, Q catheter consistently demonstrates efficient clot removal across various clot types, positioning it as a promising solution for vessel size matched ischemic stroke treatments.

## Supplemental Material

**Figure:** Video 1.

**Figure:** Video 2.

## Data Availability

The authors confirm that all data supporting the findings of this study are available within the article.
